# Risk factors of *Bartonella* spp. infection and the association between *Bartonella* spp. and T-lymphocyte subset alteration in asymptomatic retrovirus-infected cats in Bangkok Metropolitan, Thailand

**DOI:** 10.14202/vetworld.2022.2399-2406

**Published:** 2022-10-13

**Authors:** Krissda Boonaramrueng, Navapon Techakriengkrai, Channarong Rodkhum, Rosama Pusoonthornthum

**Affiliations:** 1 Department of Veterinary Medicine, Faculty of Veterinary Science, Chulalongkorn University, Bangkok, 10330, Thailand; 2 Department of Veterinary Microbiology, Faculty of Veterinary Science, Chulalongkorn University, Bangkok, 10330, Thailand; 3 Feline Health and Infectious Disease Research Unit for Excellence, Chulalongkorn University, Bangkok, 10330, Thailand

**Keywords:** *Bartonella* spp, cats, feline leukemia virus, feline immunodeficiency virus, retrovirus, risk factors, T-lymphocyte subsets

## Abstract

**Background and Aim:**

Cats are a reservoir for *Bartonella* spp. infection in humans. Human bartonellosis causes disseminated inflammation to develop in immunocompromised patients, such as those infected with human immunodeficiency virus. However, the associated risks of *Bartonella* spp. infection in immunocompromised retroviral-infected cats have been inconclusive. This study aimed to evaluate the associated risks of *Bartonella* spp. infection with the alteration of T-lymphocyte subsets of retroviral-infected cats.

**Materials and Methods:**

We collected blood samples from 161 client-owned cats at veterinary clinics and hospitals throughout the Bangkok Metropolitan area from 2017 to 2020. The samples underwent hematological biochemical tests, feline retroviral status evaluation, *Bartonella* spp. polymerase chain reaction assay, immunofluorescence assay, and CD4^+^ and CD8^+^ lymphocyte counts. Risk factors associated with *Bartonella* spp. infection were determined by odds ratio (OR). Hematological and biochemical parameters were compared using independent t-tests. CD4^+^ and CD8^+^ lymphocyte counts and the CD4^+^/CD8^+^ ratio were compared among groups classified according to their retroviral and *Bartonella* spp. infection status.

**Results:**

The prevalence of *Bartonella* spp. in our study cohort was 16.1%, and the seroprevalence was 94.9%. Cats aged >1 year were at a higher risk of seropositivity than cats aged <1 year (OR: 4.296, 95% confidence interval: 1.010–18.275). The CD8^+^ percentage was significantly higher in seropositive cats (p = 0.026). There was a significant reduction in the CD4^+^/CD8^+^ ratio between cats negative for both retrovirus and *Bartonella* spp. infection and cats with concurrent retrovirus and *Bartonella* spp. infection (p = 0.041).

**Conclusion:**

In endemic countries or areas, cat owners must be made aware of the risk of exposure to *Bartonella* spp. due to the high rate of bacteremia and seroprevalence. Retrovirus-infected cats with concurrent *Bartonella* spp. infection also showed a significant, inverted CD4^+^/CD8^+^ ratio, which may be used as a novel marker in bartonellosis. Similar studies focusing on the different stages of retrovirus infection should be undertaken further to elucidate the effect of retrovirus infection on *Bartonella* spp. infection.

## Introduction

Bartonellosis is a zoonosis caused by *Bartonella* spp. [1, 2]. Infection occurs worldwide and is more common in warm climates with high humidity [1, 3]. Human bartonellosis has various clinical presentations, of which cat scratch disease is one of the most concerning, with symptoms of mild fever and regional lymphadenopathy [4–7]. Immunocompetent individuals experience a relatively localized and self-limited lesion [8–10]. However, the disease can be debilitating and even deadly in immunocompromised patients, such as those with human immunodeficiency virus (HIV) or those who have undergone chemotherapy [7, 11, 12]. Bacillary angiomatosis and bacillary peliosis have been reported in both HIV-infected and other immunocompromised patients [11, 12]. These vascular proliferative diseases are often noted in HIV-infected adults with a low CD4+ count [13].

As companion animals, cats live closely with humans and serve as reservoirs of human bartonellosis [1, 5]. They harbor many *Bartonella* species, including *Bartonella henselae*, *B. clarridgeiae*, and *B. koehlerae* [1, 14]. Feline bartonellosis is usually subclinical and can easily spread worldwide [1, 5, 15]. Feline retroviral infections, including feline immunodeficiency virus (FIV) infection and feline leukemia virus (FeLV), are common immunosuppressive diseases in cats and are considered risk factors for clinical feline bartonellosis [16–18]. Although the mechanisms of immunosuppression differ between FeLV and FIV, they cause a reduced CD4^+^ T-lymphocyte count and an inverted CD4^+^/CD8^+^ ratio [19–22]. The relationship between bartonellosis and retroviral infections in cats has been investigated by many research teams [23, 24]. However, the association between FIV/FeLV infections and *Bartonella* spp. infection remains inconclusive.

Thus, this study aimed to estimate the prevalence and risk factors of *Bartonella* spp. infection in cats, examine the impact of retroviral infection on *Bartonella* spp. infection, and determine the effect of alterations in T-lymphocyte subsets in clinically healthy retrovirus-infected cats with concurrent *Bartonella* spp. infection.

## Materials and Methods

### Ethical approval and informed consent

This study was approved by Chulalongkorn University Animal Care and Use Committee (protocol No. 1831029, 1931953). Owner consent was obtained from each participant before the study.

### Study period and location

This cross-sectional study was conducted from the year March 2017 to November 2020 at veterinary clinics and hospitals in the Bangkok Metropolitan area. The samples were processed at the Small Animal Hospital, Chulalongkorn University, Bangkok, Thailand.

### Study population and data collection

Our study included 161 clinically healthy client-owned cats who visited for a health checkup and/or neutering. All cats underwent physical examinations by an attending veterinarian to confirm that they were clinically healthy. There were no specific criteria in terms of breed, age, or gender for enrollment in our study. We excluded cats who appeared sick with visible abnormal clinical signs and cats whose owners did not consent to study participation. We recorded the health status and clinical history, including the presence or absence of fleas, ectoparasite prevention, and lifestyle, of each cat. Flea dirt and/or visible fleas were considered evidence of flea presence. We collected 3 mL each of whole blood in ethylenediaminetetraacetic acid (EDTA), heparinized, and serum tubes to perform a complete blood count, blood chemistry tests, and feline retroviral status determination (Witness FeLV-FIV Test Kit; Zoetis, Australia). To investigate the influence of immunosuppression by retroviral infections on *Bartonella* spp. through the alteration of T-lymphocyte subsets, all cats were categorized into four groups depending on their retroviral and *Bartonella* spp. infection status as follows: Group 1, clinically healthy and negative for both *Bartonella* spp. polymerase chain reaction (PCR) and retrovirus; Group 2, negative for *Bartonella* spp. PCR but positive for retrovirus; Group 3, positive for *Bartonella* spp. PCR but negative for retrovirus; and Group 4, positive for both *Bartonella* spp. PCR and retrovirus. The CD4^+^/CD8^+^ ratios were compared among groups.

### Detection of *Bartonella* spp. DNA by PCR

We extracted DNA from the whole blood samples collected in EDTA tubes using a NucleoSpin Blood Kit (Macherey-Nagel GmbH and Co. KG, Germany). The extracted DNA was stored at −20°C until use in PCR assays. We performed nested PCR using species-specific primers targeting the 16S–23S rDNA intergenic region of *Bartonella* spp. as previously described [25]. The 16S–23S rRNA intergenic region sequence was amplified with PCR primers P-bhenfa (5'-TCTTCGTTTCTCTTTCTTCA-3') and P-benr1 (5'-CAAGCGCGCGCTCTAACC-3') and nested primers N-bhenf1a (5'-GATGATCCCAAGCCTTCTGGC-3') and N-bhenr (5'-AACCAACTGAGCTACAAGCC-3'). *Bartonella* spp. was confirmed by direct Sanger sequencing of the PCR product.

### Indirect fluorescent antibody (IFA) assay

Serological confirmation of *Bartonella* spp. was performed using a MegaFLUO BARTONELLA henselae IFA test kit (Megacor Diagnostik GmbH, Austria) as per the manufacturer’s instructions. An IFA titer ≥1:64 was considered seropositive.

### Flow cytometry analysis

The percentage of CD4^+^ and CD8^+^ T-lymphocytes and the CD4^+^/CD8^+^ ratio were examined for each cat to evaluate the immune status and the risk of secondary infection [16, 26, 27]. Briefly, 100 μL of blood collected in an EDTA tube was decanted into a 5 mL polystyrene tube. Then, the red blood cells were lysed using lysing buffer (BD Pharm Lyse, USA) as per the manufacturer’s instructions. Next, the sample was centrifuged at 200× *g* for 5 min at 4°C, and the supernatant was discarded. The remaining pellet was washed with 3 mL of wash buffer (phosphate-buffered saline [PBS] + 1% w/v bovine serum albumin [Sigma-Aldrich, UK] + 0.1% sodium azide [Sigma-Aldrich]), centrifuged at 200 × g for 5 min at 4°C, and the supernatant was discarded. The pellet was then stained with 5 μL of anti-feline CD4-FITC and anti-feline CD8-PE (Bio-Rad, USA) for 30 min at 37°C. The samples were washed once with wash buffer and centrifuged at 200× *g* for 5 min at 4°C before discarding the supernatant. The samples were preserved with the addition of 100 μL of 2% paraformaldehyde in 1% PBS and kept at 4°C until analysis. The CD4^+^ and CD8^+^ T-lymphocyte counts and the CD4^+^/CD8^+^ ratio were analyzed on a Cytomics FC 500 Flow Cytometer using CXP software (Beckman Coulter, USA).

### Statistical analysis

We used the Chi-square test to analyze *Bartonella* spp. infection and its possible risk factors, including age, presence of fleas, gender, and retroviral status. Hematological and biochemistry parameters were compared between non-infected and infected cats by independent t-tests. The percentages of CD4^+^ cells, CD8^+^ cells, and the CD4^+^/CD8^+^ ratio were measured and compared between the groups that were positive and negative for *Bartonella* spp. infection, and antibody levels against *Bartonella* spp. were compared using the Mann–Whitney U-test. The Kruskal–Wallis test and *post hoc* analysis were employed to compare the *Bartonella* spp.-infected and retrovirus-infected groups. p < 0.05 was considered statistically significant. The odds ratio (OR) was considered statistically significant if the 95% confidence interval did not exceed 1.0.

## Results

**Figure-1:**
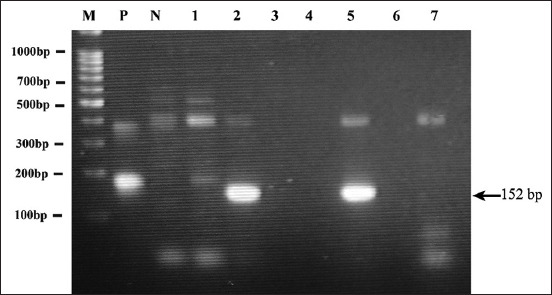
Identification of *Bartonella* spp. by a nested polymerase chain reaction in 16S-23S rDNA intergenic regions. M lane is DNA ladder. P lane is positive control showed expected band at 152 bp fragment for *Bartonella henselae* (arrow). N lane is negative control. Lanes 2 and 5 showed positivity.

Our study cohort included 161 clinically healthy cats, of which 73 (45.3%) were male and 88 (54.7%) were female. The median age was 1.5 years (range: 2 months–12 years). Among the 27 cats who were positive for a feline retrovirus, 11 (6.8%) tested positive for FIV, 18 (11.2%) tested positive for FeLV, and two tested positive for both FeLV and FIV. There were 26 positive cats (16.1%) and 135 negative cats (83.9%) for *Bartonella* spp. by nested PCR (Figure-1). However, the presence of antibodies against *Bartonella* spp. detected by IFA was high, with 150 (94.9%) cats testing positive and 8 (5.1%) testing negative.

The mean ± SD was 14,231.21 ± 5.757 cells/μL for white blood cells, 34.78 ± 7.71% for hematocrit, and 38,883 ± 7,515 cells/μL for platelets. Biochemistry tests revealed that the mean ± SD for urea nitrogen and creatinine was 28.18 ± 12.92 and 1.45 ± 0.68 mg/dL, respectively, and that of serum alanine aminotransferase and alkaline phosphatase was 75.91 ± 74.50 and 42.67 ± 31.71 units/l, respectively. The mean ± SD for serum total protein, albumin, and globulin was 7.51 ± 0.83, 2.90 ± 0.33, and 4.61 ± 0.90 mg/dL, respectively (Table-1).

**Table-1:** Complete blood count, blood chemistry, *Bartonella* spp. PCR result, and number of cats in each category.

Parameters	Mean ± SD	*Bartonella* spp. PCR		p*-*value

		Negative Mean ± SD	Positive Mean ± SD	
WBC (×10^3^cells/μL)	14.23 ± 5.76	14.09 ± 5.90 n = 128	14.91 ± 5.03 n = 26	0.51
HCT (%)	34.78 ± 7.71	35.13 ± 7.96 n = 128	33.03 ± 6.21 n = 26	0.206
PLT (×10^3^cells/μL)	138.88 ± 75.15	137.34 ± 76.72 n = 128	146.5 ± 67.80 n = 26	0.573
BUN (mg/dL)	28.18 ± 12.92	28.36 ± 13.89 n = 131	27.22 ± 5.68 n = 25	0.687
Creatinine (mg/dL)	1.45 ± 0.68	1.45 ± 0.73 n = 132	1.46 ± 0.27 n = 26	0.970
Total protein (mg/dL)	7.51 ± 0.83	7.51 ± 0.86 n = 126	7.55 ± 0.66 n = 24	0.838
Albumin (mg/dL)	2.90 ± 0.33	2.91 ± 0.34 n = 126	2.90 ± 0.25 n = 24	0.859
Globulin (mg/dL)	4.61 ± 0.90	4.61 ± 0.94 n = 124	4.65 ± 0.67 n = 24	0.832
ALT (units/L)	75.91 ± 74.50	78.39 ± 79.06 n = 132	63.35 ± 43.59 n = 26	0.348
ALP (units/L)	42.67 ± 31.71	41.51 ± 30.44 n = 125	48.96 ± 38.03 n = 23	0.302

*Significance difference when *p* <0.05. WBC=White blood cell count, HCT=Hematocrit, PLT=Platelet count, BUN=Blood urea nitrogen, ALT=Serum alanine aminotransferase, ALP=Serum alkaline phosphatase, SD=Standard deviation, PCR=Polymerase chain reaction

There were no significant differences between *Bartonella* spp. PCR status and total white blood cell count, hematocrit, platelet count, blood urea nitrogen, creatinine, total protein, albumin, globulin, alanine aminotransferase, and alkaline phosphatase (Table-1). Cats aged >1 year were significantly associated with *Bartonella* spp. seropositivity (OR: 4.296) (Table-2). However, the cats’ ages were unrelated to *Bartonella* spp. PCR status (OR: 2.480). Feline retroviral infections (FeLV or FIV positive), gender, and flea infestation status were not associated with either *Bartonella* spp. serostatus or *Bartonella* spp. PCR status (Tables-2 and 3).

**Table-2:** Association (odds ratio) between gender, age, flea infestation, and retrovirus status, with IFA results of *Bartonella* spp. antibody status.

Variables	*Bartonella* spp. Ab status		OR	95% CI	
	
	Negative n (%)	Positive n (%)		Lower	Upper
Gender (n = 158)					
Male	1 (1.4)	70 (98.6)	0.163	0.020	1.360
Female	7 (8.0)	80 (92.0)			
Age (n = 158)					
<1 year	4 (11.1)	32 (88.9)	4.296	1.010	18.275
≥1 year	4 (3.3)	118 (96.7)			
Fleas (n = 158)					
No	5 (5.4)	87 (94.6)	1.207	0.278	5.237
Yes	3 (4.5)	63 (95.5)			
Retroviral status (n = 158)					
Negative	8 (6.1)	123 (93.9)	ND	ND	ND
Positive	0 (0)	27 (100)			

OR=Odds ratio, 95% CI=95% confidence interval, SD=Standard deviation, Fleas=Flea infestation, Ab=Antibody, ND=Not determined, IFA=Indirect fluorescent antibody

**Table-3:** Association (odds ratio) between gender, age, flea infestation, retrovirus status, and antibody status with *Bartonella* spp. PCR results.

Variables	*Bartonella* spp. PCR		OR	95% CI	
	
	Negative n (%)	Positive n (%)		Lower	Upper
Gender (n = 161)					
Male	61 (83.6)	12 (16.4)	0.962	0.414	2.233
Female	74 (84.1)	14 (15.9)			
Age (n = 161)					
<1 year	33 (91.7)	3 (8.3)	2.480	0.700	8.793
≥1 year	102 (81.6)	23 (18.4)			
Fleas (n = 161)					
No	84 (88.4)	11 (11.6)	2.246	0.958	5.267
Yes	51 (77.3)	15 (22.7)			
Retroviral status (n = 161)					
Negative	112 (83.6)	22 (16.4)	0.885	0.279	2.813
Positive	23 (85.2)	4 (14.8)			
Ab status (n = 158)					
Negative	7 (87.5)	1 (12.5)	1.400	0.165	11.885
Positive	125 (83.3)	25 (16.7)			

OR=Odds ratio, 95% CI=95% confidence interval, SD=tandard deviation, Fleas=Flea infestation, Ab=Antibody, PCR=Polymerase chain reaction

**Figure-2:**
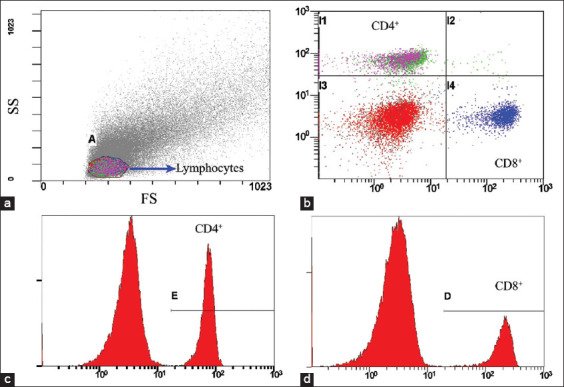
Representative flow cytometry analysis of CD4^+^ and CD8^+^ lymphocytes. (a) Forward scatter height (FS, X-axis) was plotted against side scatter height (SS, Y-axis) to select a lymphocyte population. (b) CD8^+^ PE (CD8^+^, X-axis) was plotted against CD4^+^ FIT-C (CD4^+^, Y-axis). (c and d) are histograms of CD4^+^ and CD8^+^ T-lymphocytes, respectively.

The median values of CD4^+^ and CD8^+^ T-lymphocytes were 15.175% (range: 1.28%−48.46%) and 8.350% (range: 1.24%–41.88%), respectively. The median CD4^+^/CD8^+^ ratio was 1.853 (range: 0.323−5.631). Figure-2 shows a representative flow cytometry analysis. The median CD8^+^ percentage of FIV-negative cats was significantly lower than FIV-positive cats (p = 0.026). In addition, the median CD4^+^/CD8^+^ ratio of FIV-negative cats was significantly higher than FIV-positive cats (p = 0.045). The percentage of CD8^+^ cells in seronegative cats was significantly lower than in seropositive cats (p *=* 0.024) (Table-4).

**Table-4:** Median of CD4^+^ and CD8^+^ T-lymphocyte, and CD4^+^ to CD8^+^ ratio for each category.

Cat’s status		CD4+ (%)	Range	p-value	CD8+ (%)	Range	p-value	CD4:8 ratio	Range	p-value
All	n = 106	15.175	1.28–48.46	ND	8.350	1.24–41.88	ND	1.853	0.323–5.631	ND
FIV	Negative (n = 99)	15.710	1.28–48.86	0.099	8.100	1.24–41.88	0.026*	1.896	0.323–5.065	0.045*
	Positive (n = 7)	12.120	7.38–16.16		14.300	6.38–26.57		1.119	0.633–5.631	
FeLV	Negative (n = 92)	15.050	1.28–48.86	0.955	8.350	1.24–32.28	0.346	1.859	0.455–5.631	0.083
	Positive (n = 14)	15.415	2.38–29.58		9.720	2.67–41.88		1.644	0.323–3.306	
*Bartonella* spp. PCR	Negative (n = 85)	15.040	1.28–48.86	0.778	8.310	1.24–32.28	0.269	1.903	0.455–5.631	0.338
	Positive (n = 21)	15.290	4.07–26.02		8.770	2.46–41.88		1.654	0.323–4.000	
*Bartonella* spp. antibody	Negative (n = 100)	7.510	5.67–24.03	0.229	5.495	2.08–7.25	0.024*	2.705	1.088–2.667	0.251
	Positive (n = 6)	15.415	1.28–48.86		8.740	1.24–41.88		1.806	0.323–5.631	

* Significance difference when p*<*0.05. FIV: Feline immunodeficiency virus (witness, Zoetis), FeLV=Feline leukemia virus (witness, Zoetis), Neg=Negative, Pos=Positive, ND=Not determined, PCR=Polymerase chain reaction

**Figure-3:**
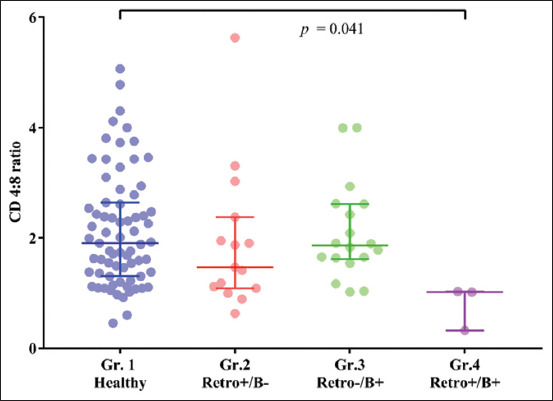
Column scatter plot of CD4^+^ to CD8^+^ ratios in four groups of cats with different retrovirus and *Bartonella* spp. statuses. Gr.1: Healthy cats who tested negative for both *Bartonella* spp. Polymerase chain reaction (PCR) and retrovirus; Gr.2: Cats who tested negative for *Bartonella* spp. PCR but positive for retrovirus; Gr.3: Cats who tested positive for *Bartonella* spp. PCR but negative for retrovirus; and Gr.4: Cats who tested positive for both *Bartonella* spp. PCR and retrovirus.

The cats were divided into four groups according to their retrovirus and *Bartonella* spp. PCR results to compare the CD4^+^/CD8^+^ ratio. The Kruskal–Wallis test revealed a significant difference among groups (p = 0.034). All pairwise *post hoc* analyses showed the lowest CD4^+^/CD8^+^ ratio in Group 4 (median: 1.017, range: 0.323−1.027), which was significantly lower than Group 1 (median: 1.914, range: 0.455−5.065) (p = 0.041) but not significantly lower than Group 2 (median: 1.469, range 0.633−5.631) (p = 0.308) or Group 3 (median: 1.864, range 1.021−4.001) (p = 0.056) (Figure-3).

## Discussion

This study evaluated the risk of exposure to bartonellosis from cats living in the Bangkok Metropolitan area and the influence of feline retroviral infection on *Bartonella* spp. through the alteration of T-lymphocyte subpopulations. In Bangkok, where temperature and humidity remain high throughout the year, 16.1% of the cats in our study showed positive *Bartonella* spp. PCR results and most (94.9%) were seropositive. These results indicate that most cats in the Bangkok Metropolitan area were previously exposed to *Bartonella* spp. at some point in time. A high seroprevalence (up to 93%) in free-roaming cats has been reported in other studies [13, 28–32]. The prevalence in our study is higher than in other countries, which suggests that the weather in Thailand, a Southeast Asia country, promotes the risk of infection. These findings also mean that the risk of exposure of cat owners to *Bartonella* spp. is high, especially in the Bangkok Metropolitan area. A higher risk of *Bartonella* spp. bacteremia or DNA detection has been reported in young cats [1, 14, 30]. However, the rate of seropositivity in our study was lower in younger cats than in older cats. It should be noted that fluctuating bacteremia in cats can cause undetectable levels, even when there is persistent bacteremia [33]. Moreover, our results did not show any association with hematological findings in *Bartonella* spp.-infected cats, which was consistent with the previous studies [1, 34, 35].

Bartonellosis symptoms are dependent on host immunity [13, 15]. The HIV-positive or immunocompromised people may develop severe clinical conditions, especially in HIV patients with a low CD4^+^ count [7, 13, 15, 36]. Feline retrovirus infections (FeLV and FIV) in cats lead to immunocompromisation [21]. In cats experimentally infected with *B. henselae*, the number of CD4^+^ T-lymphocytes decreased after infection, and interferon-γ and tumor necrosis factor-α were identified as important keys in eliminating *B. henselae* infection [27]. However, instead of interferon-γ, increased interleukin-4 production was found in naturally infected cats. This phenomenon may result in the relapsing bacteremia observed in naturally infected cats [37, 38]. However, there was no clinical relationship between *Bartonella* spp. and retroviral infection in our study or others [14, 24, 39]. The retroviral infection stages could explain this phenomenon. Cats with terminal stage FIV may suffer from opportunistic infections [40]. Similar observations of decreased ratios CD4^+^/CD8^+^ ratios were reported in FIV-positive cats with *Toxoplasma gondii* infection [41] and FIV-positive cats with cutaneous fungal colonization [42]. These concurrent infections are the result of the decreased CD4^+^/CD8^+^ ratio [41–43]. These decreased CD4^+^/CD8^+^ ratios were also reported in retrovirus-infected cats and *Bartonella* spp. coinfected cats in our study. In addition, cats with concurrent retroviral and *Bartonella* spp. infection had the lowest CD4^+^/CD8^+^ ratio in our investigation. These data may support the hypothesis that the host response to *Bartonella* spp. infection includes humoral and cell-mediated mechanisms that may be disrupted by a feline retrovirus, thereby underlining the lowest CD4^+^/CD8^+^ ratio in our study.

Our study had some limitations. First, the cats in our study cohort may have had undetected concurrent illnesses that altered their T-lymphocyte subpopulations. Second, the retrovirus-infected cats, especially those that were FIV positive, were not classified according to the stage of FIV infection because of the low number of FIV-positive cats. Further investigation of CD expression at different stages of FIV infection should be undertaken to elucidate the effect of CD4^+^ and CD8^+^ levels, as well as the CD4^+^/CD8^+^ ratio on *Bartonella* spp. infection.

## Conclusion

The high rate of bacteremia and seroprevalence in the cats in this study demonstrates the significant zoonotic potential of *Bartonella* spp. in cats in the Bangkok Metropolitan area. The awareness of cat owners of the risk of exposure to *Bartonella* spp. should also be promoted in other warm and humid countries and in endemic areas. Our investigation of retrovirus-infected cats showed the significant role of the host response to the *Bartonella* spp. infection. Furthermore, an inverted CD4^+^/CD8^+^ ratio should be proposed as a novel marker for bartonellosis.

## Authors’ Contributions

KB, NT, CR, and RP: Conceived and designed the study. KB and NT: Performed the experiments and analyzed the data. NT and CR: Contributed reagents, materials, and analysis tools. KB, NT, CR, and RP: Wrote and edited the manuscript. All authors have read and approved the final manuscript.
